# Antihypertensive drug concentration measurement combined with personalized feedback in resistant hypertension: a randomized controlled trial

**DOI:** 10.1097/HJH.0000000000003585

**Published:** 2023-10-18

**Authors:** Laura E.J. Peeters, M.H.W. Kappers, D.A. Hesselink, J.B. van der Net, S.C.C. Hartong, R. van de Laar, M. Ezzahti, P.J.G. van de Ven, I.M. van der Meer, E.L.E. de Bruijne, A.A. Kroon, S. Indhirajanti-Tomasoa, N.A.J. van der Linde, S. Bahmany, E. Boersma, E. K. Massey, L. van Dijk, T. van Gelder, Birgit C.P. Koch, Jorie Versmissen

**Affiliations:** a Erasmus MC, University Medical Center Rotterdam, Department of Hospital Pharmacy; b Erasmus MC, University Medical Center Rotterdam, Department of Internal Medicine, Rotterdam; c Amphia Hospital, Department of Internal Medicine, Breda; d Albert Schweitzer Hospital, Department of Internal Medicine, Dordrecht; e Ikazia Hospital, Department of Internal Medicine, Rotterdam; f Bravis Hospital, Department of Internal Medicine, Bergen op Zoom; g Maasstad Hospital, Department of Internal Medicine, Rotterdam; h HAGA Hospital, Department of Internal Medicine, The Hague; i IJsselland Hospital, Department of Internal Medicine, Capelle aan den Ijssel; j Maastricht University Medical Center, Department of Internal Medicine, Maastricht; k Franciscus gasthuis & Vlietland, Department of Internal Medicine, Rotterdam; l Reinier de Graaf Hospital, Department of Internal Medicine, Delft; m Erasmus MC, University Medical Center Rotterdam, Department of Cardiology, Rotterdam; n Nivel, Netherlands Institute for Health Services Research, Department Pharmaceutical Care, Utrecht; o Unit of PharmacoTherapy, Epidemiology & Economics, Groningen Research Institute of Pharmacy, University of Groningen, Groningen, The Netherlands

**Keywords:** adherence, antihypertensive drugs, hypertension, intervention, therapeutic drug monitoring

## Abstract

**Background:**

Adherence to antihypertensive drugs (AHDs) is crucial for controlling blood pressure (BP). We aimed to determine the effectiveness of measuring AHD concentrations using a dried blood spot (DBS) sampling method to identify nonadherence, combined with personalized feedback, in reducing resistant hypertension.

**Methods:**

We conducted a multicenter, randomized, controlled trial (RHYME-RCT, ICTRP NTR6914) in patients with established resistant hypertension. Patients were randomized to receive either an intervention with standard of care (SoC) or SoC alone. SoC consisted of BP measurement and DBS sampling at baseline, 3 months (t3), 6 months (t6), and 12 months (t12); AHD concentrations were measured but not reported in this arm. In the intervention arm, results on AHD concentrations were discussed during a personalized feedback conversation at baseline and t3. Study endpoints included the proportion of patients with RH and AHD adherence at t12.

**Results:**

Forty-nine patients were randomized to receive the intervention+SoC, and 51 were randomized to receive SoC alone. The proportion of adherent patients improved from 70.0 to 92.5% in the intervention+SoC arm (*P* = 0.008, *n* = 40) and remained the same in the SoC arm (71.4%, *n* = 42). The difference in adherence between the arms was statistically significant (*P* = 0.014). The prevalence of resistant hypertension decreased to 75.0% in the intervention+SoC arm (*P* < 0.001, *n* = 40) and 59.5% in the SoC arm (*P* < 0.001, *n* = 42) at t12; the difference between the arms was statistically nonsignificant (*P* = 0.14).

**Conclusion:**

Personalized feedback conversations based on DBS-derived AHD concentrations improved AHD adherence but did not reduce the prevalence of RH.

## Graphical abstract

**Figure:**
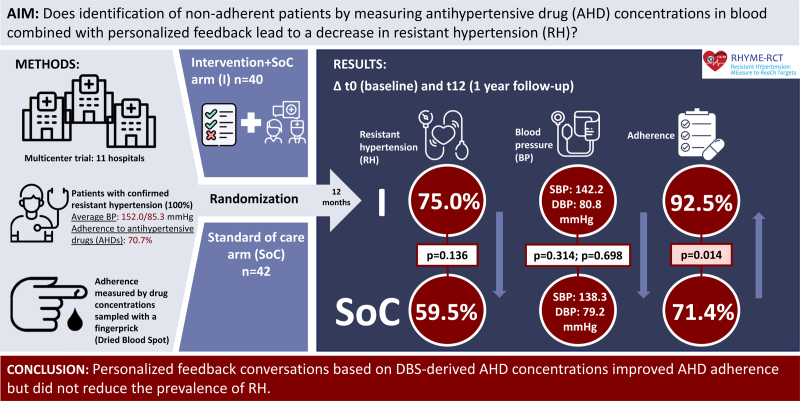

## INTRODUCTION

Nonadherence to antihypertensive drugs (AHDs) is one of the most important barriers to achieve blood pressure control [1,2]. Uncontrolled blood pressure and thereby resistant hypertension, or in case of nonadherence “pseudo-RH,” is associated with an increased risk of coronary heart disease and end-stage kidney disease. However, both identifying and reducing nonadherence remain challenging. Correct identification of nonadherent patients in clinical practice by healthcare providers is suboptimal [3]. The most precise approach for detecting nonadherence is to measure drug concentrations in blood, despite being considered invasive under the regulations of the Medical Research Involving Human Subjects acts. This makes it also the most invasive method among various options for assessing nonadherence [4,5]. In order to enhance the usability of measuring drug concentrations in blood while simultaneously reducing the challenges associated with venipuncture, we concentrated on improving the method of measuring drug concentrations in blood. This improvement involved employing a previously validated dried blood spot (DBS) sampling technique, which requires a fingerprick [6,7].

Only a few randomized clinical trials have been performed that investigate interventions improving adherence in patients with resistant hypertension [8–10]. The positive effect of behavioral interventions combined with measuring AHD concentrations in hypertensive patients was already shown in observational studies, but these retrospective studies did not include a standardized method to implement the behavioral intervention [11,12].

In this unique randomized, controlled trial RHYME-RCT (Resistant HYpertension: MEasure to ReaCh Targets), we compared an intervention with DBS sampling at the moment of blood pressure measurement combined with feedback using our communication tool on top of standard of care (SoC) compared with SoC alone. The primary aim of our trial was to determine whether this intervention leads to a decrease in resistant hypertension. Our second aim was to determine whether this decrease in resistant hypertension was due to an improvement in adherence.

## MATERIALS AND METHODS

RHYME-RCT (ICTRP, NTR6914, https://trialsearch.who.int/Trial2.aspx?TrialID=NTR6914) is a randomized, multicenter, single-blinded, controlled trial to improve adherence to AHDs and thereby improve blood pressure and resistant hypertension. Reporting is based on the ESPACOMP Medication Adherence Reporting Guidelines (EMERGE) and CONSORT reporting guidelines [13,14]. The extensive version of our protocol was previously published and can be consulted for details on our methods section [15].

### Participants

Patients were recruited at the vascular, cardiology, and nephrology departments of 12 hospitals in the Netherlands of which two were tertiary centers. Patients were eligible if they were at least 18 years old, used three AHDs including a diuretic or four AHDs of which at least two could be measured with a validated ultra-high performance liquid chromatography-tandem mass spectrometry (UHPLC-MS/MS) method, and had an office blood pressure or ambulatory office blood pressure measurement (AOBP) of at least 140 and/or 90 mmHg. The UHPLC-MS/MS method included AHDs that were most prescribed in the recruiting hospitals and included 12 AHDs and four of their [active metabolites]: enalapril and [enalaprilate], perindopril and [perindoprilate], irbesartan, valsartan, losartan and [losartan-carboxylic acid (losartan-CA)], hydrochlorothiazide, bumetanide, spironolactone and [canrenone], amlodipine, barnidipine, nifedipine, metoprolol, and doxazosin [6,16].

Exclusion criteria were an estimated GFR of 15 ml/min per m^2^ or less, possible secundary causes of hypertension that were not excluded yet, unwillingness to do a 24-h ambulatory blood pressure measurement (24-h ABPM) or insufficient understanding of the Dutch or Turkish language to read the patient information leaflet. It was not allowed to make any changes in AHD therapy between the selection of patients and the eligibility visit.

Before participation patients had to provide written informed consent. This study was approved by the local medical ethical committee of the Erasmus Medical Center, Rotterdam, the Netherlands (MEC-2018–027).

### Study design

After signing informed consent, patients went to an eligibility visit where blood was drawn using DBS sampling and simultaneously a 24-h ABPM was started to measure blood pressure in the following 24 h. The 24-h ABPM was obliged to minimize white-coat hypertension.

If daytime blood pressure measured with ABPM was at least 135 and/or 85 mmHg, patients were randomized in a 1 : 1 ratio to either a SoC arm or intervention+SoC arm using a digital randomization tool (ALEA) [15].

SoC consisted of visits at 3 (t3), 6 (t6), and 12 (t12) months after the eligibility visit and included a blood pressure measurement, DBS sampling, and a visit to an internist or nurse specialist. All blood pressure measurements were performed as indicated in the current American and European hypertension guidelines [15,17,18]. Results on the blood pressure measurements were available in this arm, but no results on the actual adherence based on drug concentrations were reported to healthcare providers.

Directly after randomization, before any results on adherence were available, the treating physician or nurse was asked to estimate the adherence rate of included patients, to determine if there was any bias in selecting patients.

#### Intervention

The intervention consisted of a comprehensive feedback conversation at baseline and t3 where adherence results were discussed with the help of a communication tool [19] independently of the actual results (adherent or nonadherent). At t6 and t12, adherence results were reported to the treating physician or nurse specialist, but they were not allowed to discuss this with the patient. This was done to see if the intervention endured over time. Because of the structure of this intervention, patients were only allowed to miss a single visit at either t3 or t6 to at least include the two intervention visit and one visit to see endurance of the intervention. If patients missed more than two visits, they were excluded from the per-protocol analysis.

#### Adherence

Adherence was measured using drug concentrations in blood sampled with a DBS and measured with UHPLC-MS/MS. Sampling of blood was always combined with a blood pressure measurement at the same time. Measured drug concentrations were either reported as positive, present in blood, or negative, absent in blood. To minimize false-negative outcomes, only undetected AHDs were reported as negative. The intra-patient variability of AHD concentrations in blood is high, and therefore, trough levels could not be used as a cut-off for nonadherence [7,20]. Furthermore, only if both the parent drug and metabolite could not be detected in blood, this was reported as negative. All measured AHDs were detectable at least 24 h after intake with the exception of hydrochlorothiazide.

Patients were categorized as nonadherent if all measured AHDs were reported as negative, partially nonadherent if one or more AHDs were reported as negative but at least one AHD was reported as positive, and adherent if all measured AHDs were reported as positive. The adherence data were converted into a binary variable by combining total nonadherent and partially nonadherent together.

#### COVID-19

Due to COVID-19 and a temporary reduction of physical visits at the outpatient clinics, some changes had to be made with regard to the visit schedule of randomized patients to maintain a total of three and preferable four visits. When the closing visit at t12 was missed, this was rescheduled at the first convenient moment in time to stay as close as possible to a 12 months follow-up. Because of this change in schedule, data were analyzed in accordance with their visit number: visit 1 = t0, visit 2 = t3, visit 3 = t6, and visit 4 = t12.

#### Sample size calculation

The sample size calculation is based on the expected improvements in the prevalence of resistant hypertension due to improvements in adherence (Supplemental material Figure S1). It was expected that the prevalence of resistant hypertension at t12 improved (i.e. reduced) from 100 to 80% in the intervention+SoC arm due to the intervention and participation in a study and from 100 to 90% in the SoC alone due to participation in the study. The extent of the improvements was based on several previously conducted adherence trials [12,21,22]. A total of 392 patients were required to demonstrate a difference of 10% between both arms after 12 months of follow-up with a β = 0.8 (power = 80%) and a two-sided α = 0.05 [15].

### Data analysis

Continuous variables with a normal distribution are described as mean value ± one standard deviation (SD), and otherwise as median value (25th to 75th percentile). Normality was tested by the Shapiro--Wilk test. Categorical variables are described as numbers and percentages. Within-group changes in percentage adherence and resistant hypertension from t0 to t12 were evaluated by McNemar tests. We used a chi^2^-test to study differences in adherence and resistant hypertension between patients randomized to the intervention+SoC versus SoC alone. For this, we tested the arms both separate from each other by selecting patients based on their randomization arm and as a whole group.

Differences in mean blood pressure over time, t0 versus t12, within the same arm were tested using a paired sample *t*-test and differences between patients randomized to the intervention+SoC versus SoC alone with an independent samples *t*-test.

We performed an intention-to-treat analysis, including all randomized patients, as well as a per-protocol analysis, including the patients with available measurements at t0 and t12, and one of t3 and t6. The per-protocol analysis is preferred while all steps [visit 1: first feedback conversation, visit 2 (t3 or t6): repeated feedback conversation and visit 3/4: follow-up] of the intervention are included.

To determine the influence of adherence on resistant hypertension (dependent variable) generalized estimating equations (GEEs) were used, which could account for the clustering of data within individuals due to multiple visits. The calculated defined daily dose (DDD) at t0 and t12 as well as the randomization arm were included as covariates in this binary regression model.

All *P* values were two-sided, and a value of less than 0.05 was considered statistically significant.

We used the SPSS version 24.0 for Windows (IBM Corp, Armonk, New York, USA), and GraphPad Prism 9.3 software (GraphPad Software, La Jolla, California, USA) for analysis.

### Interim analysis

An interim analysis was performed with data from the trial to check the assumptions made in the sample size calculation. For this, at least 25 patients had to be included in both arms that had reached three months follow-up. We expected to find a larger difference between the arms in the proportion of patients with resistant hypertension at this time point due to the shorter follow-up time, as compared to results after 12 months of follow-up. At the time of the interim analysis, 27 patients in the intervention+SoC arm and 28 patients in the SoC arm were analyzed. In the intervention+SoC arm, the proportion of patients with resistant hypertension decreased to 75% and in the SoC arm to 59%. This decrease in resistant hypertension was higher than expected. However, there was a reversed improvement in resistant hypertension, with a difference between the two arms of 16% in the proportion of patients with resistant hypertension with a lower proportion of resistant hypertension in the SoC arm as compared to the intervention+SoC arm. Given these interim findings and difficulties with the inclusion and visits of patients due to COVID-19 restrictions, the inclusion of patients was stopped by the researchers before reaching the required number of patients to determine the efficacy of the intervention. However, because of ethical considerations, the complete protocol was carried out for patients already included in the trial.

## RESULTS

Patients were recruited from August 2018 till January 2021.

### Patient characteristics

**FIGURE 1:**
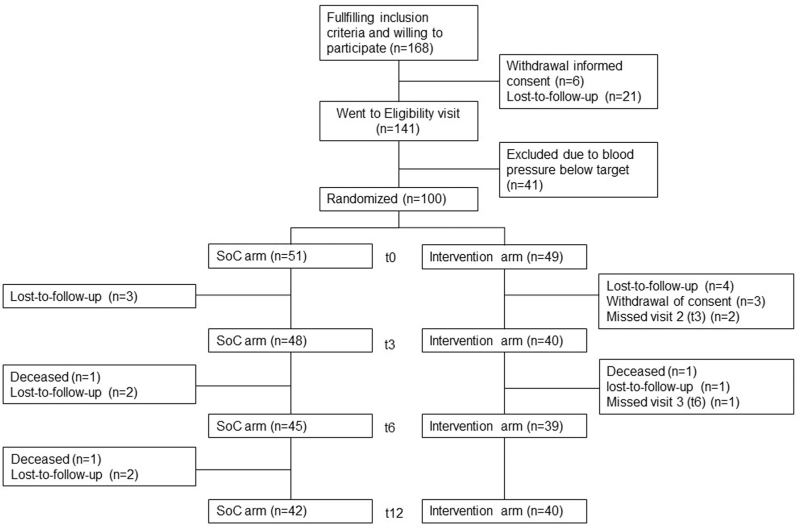
Inclusion flowchart RHYME-RCT.

A flowchart of the 168 patients that intended to participate in the trial is presented in Fig. 1. In total, 100 patients were included and randomized: 51 to the intervention+SoC arm and 49 to SoC alone arm (Table 1). The mean age was 59.4 ± 11.1 years, 70% was male, and the mean SBP/DBP was 152/86 mmHg. During the trial, 25 serious adverse events (SAEs) were registered including three patients who died due to underlying diseases including kidney failure and cardiovascular events (Fig. 1). None of the SAEs were related to the actual intervention.

**TABLE 1:** Baseline characteristics of patients measured as part of the RHYME-RCT trial

		Randomized patients (*n* = 100)		
	Excluded patients (*n* = 41)	Total	SoC alone arm (*n* = 51)	Intervention+SoC arm (*n* = 49)
Male, *n* (%)	27 (65.9)	70 (70.0)	35 (68.6)	35 (71.4)
Age (years)	59.6 ± 12.3	59.4 ± 11.1	58.1 ± 12.0	60.7 ± 10.0
BMI (kg/m^2^)	31.5 ± 5.3	30.3 ± 5.2	30.4 ± 5.6	30.2 ± 4.8
CKD-EPI eGFR (ml/min per 1.73 m^2^)	69.0 (45.5–82.0)	75.0 (48.0–90.0)	80.0 (52.0–90.0)	70.0 (46.0–89.0)
Creatinine (μmol/l)	116.5 ± 55.3	109.4 ± 57.5	108.9 ± 66.6	109.9 ± 46.9
Mean 24-h SBP (mmHg) missing, *n*	125.4 ± 10.1	148.1 ± 15.3 7^∗^	147.2 ± 16.1 3^∗^	149.1 ± 14.8 4^∗^
Mean 24-h DBP (mmHg)	72.6 ± 8.3	83.1 ± 2.8	83.2 ± 13.3	83.0 ± 12.6
Mean daytime SBP (mmHg)	125.3 ± 10.0	151.1 ± 15.3	150.2 ± 15.6	152.0 ± 15.0
Mean daytime DBP (mmHg)	75.0 ± 9.5	85.7 ± 13.4	86.1 ± 14.2	85.3 ± 12.8
Mean night-time SBP (mmHg) missing, *n*	119.8 ± 13.3	142.0 ± 18.4 8^∗^	142.2 ± 20.0 3∗	141.9 ± 16.8 5^∗^
Mean night-time DBP (mmHg)	67.3 ± 9.3	77.4 ± 13.0	77.7 ± 13.6	77.2 ± 12.4
Average usable BP recordings (%)	72.0	77.4	76.4	78.5
Adherence rate at baseline (*t* = 0) (%)	78.0	68.0	69.0	67.0
Diabetes mellitus, *n* (%)	19 (46.3)	37 (37.0)	20 (39.2)	17 (34.7)
Myocardial infarction, *n* (%)	10 (24.4)	21 (21.0)	7 (13.7)	14 (28.6)
Stroke, *n* (%)	5 (12.2)	11 (11.0)	5 (9.8)	6 (12.2)
Atrial fibrillation, *n* (%)	5 (12.2)	9 (9.0)	3 (5.9)	6 (12.2)
Heart failure, *n* (%)	1 (2.4)	4 (4.0)	4 (7.8)	0
Hypercholesterolemia, *n* (%)	11 (26.8)	38 (38.0)	20 (39.2)	18 (36.7)
Asthma/COPD, *n* (%)	4 (9.8)	13 (13.0)	6 (11.8)	7 (14.3)
Mean number of used drugs, *n*	9.7 ± 4.0	10.2 ± 4.7	9.5 ± 4.0	10.9 ± 5.4
Mean number of used AHDs, *n*	4.0 ± 0.9	4.28 ± 1.0	4.4 ± 0.8	4.2 ± 1.1
Number of patients with number of measured AHDs, *n*				
≤2	17	41	18	23
3+4	23	54	30	24
≥5	1	5	3	2
Mean DDD of used AHDs	5.4 ± 0.9	6.4 ± 2.1	6.6 ± 1.9	6.2 ± 2.2
Average measured AHDs of total used AHDs (%)	71.2	68.2	69.5	66.8
Groups used AHDs , *n* (%)				
ACEi	15 (36.6)	45 (45.0)	26 (51.0)	19 (38.8)
ARBs	22 (53.7)	54 (54.0)	26 (51.0)	28 (57.1)
Beta-blockers	30 (73.2)	61 (61.0)	28 (54.9)	33 (67.3)
Calcium antagonists	37 (90.2)	89 (89.0)	47 (92.2)	42 (85.7)
Diuretics	41 (100.0)	90 (90.0)	48 (94.1)	42 (85.7)
Other incl. doxazosin	11 (26.8)	45 (45.0)	22 (43.1)	23 (46.9)

Shown values are mean with standard deviation. eGFR is shown as median with IQR (25–75 percentile). ACEi, angiotensin-converting enzyme inhibitors; AHD, antihypertensive drug; ARB, angiotensin receptor blocker; CKD-EPI, Chronic Kidney Disease Epidemiology Collaboration; COPD, chronic obstructive pulmonary disease; DDD, defined daily dose; eGFR, estimated glomerular filtration rate; IQR, interquartile range; SoC, Standard of Care. ^a^These patients had a previous 24-h ABPM within two weeks prior to the study measurements, meeting hypertension specifications according to the study protocol. However, for the comparison with their adherence data, an office blood pressure was employed.

### Blood pressure

**FIGURE 2:**
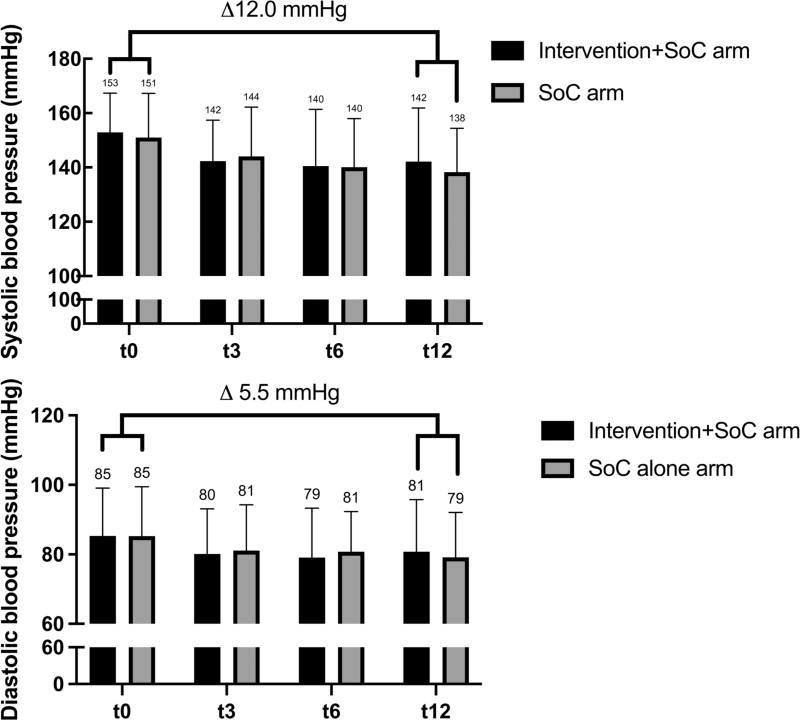
Proportion of patients with resistant hypertension (*n* = 82) at four time points. ^a^Only patients with t0, t3, or t6 and t12 are included in this figure.

Blood pressure in both arms decreased over time (Fig. 2 and Supplemental Material Figure S2). The mean blood pressure at t12 in the intervention+SoC and SoC arms was similar: 142.2/80.8 and 138.3/79.2, respectively. Both arms combined, the mean decrease in SBP was 12.0 mmHg [95% confidence interval (95% CI 7.4--16.2; *P* < 0.001], and DBP 5.5 mmHg (95% CI 2.7--7.9; *P* < 0.001). Note that there is a slight discrepancy in blood pressure averages between Table 1 and Fig. 2 due to the use of a per-protocol analysis for changes over time.

### Resistant hypertension

**FIGURE 3:**
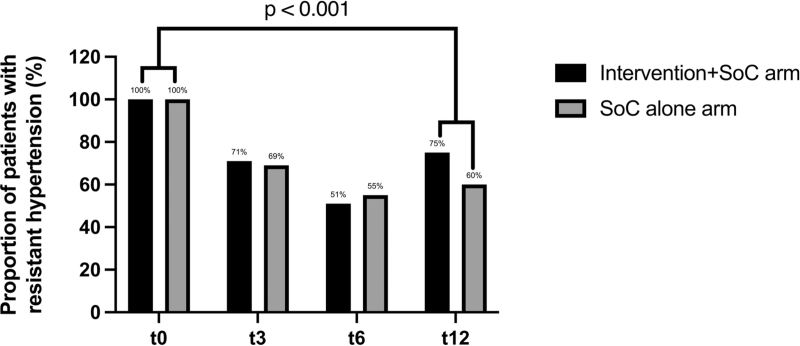
SBP and DBP at four time points (*n* = 82). ^a^Only patients with t0, t3 or t6 and t12 are included in this figure.

The proportion of patients with resistant hypertension decreased over time with the largest change between t0 and t3 (intervention+SoC arm Δ −28.9%, SoC arm Δ −31.0%) (Fig. 3). This decrease in resistant hypertension was sustained at t6 in both arms, but the proportion of patients with resistant hypertension increased again between t6 and t12 (intervention+SoC arm Δ +23.7%, SoC arm Δ +4.7%).

At 12 months follow-up, no statistically significant difference in the proportion of patients with resistant hypertension was observed between the arms (75.0 versus 59.5%, *P* = 0.14). When combining patients from both arms, a significant decrease in resistant hypertension (Δ −34.8%) was found between t0 and t12 (*P* < 0.001, *n* = 82).

No differences were found in outcomes for the per-protocol and intention-to-treat analysis. Therefore, only the results of the per-protocol analysis are shown (*n* = 82).

### Adherence rate

**FIGURE 4:**
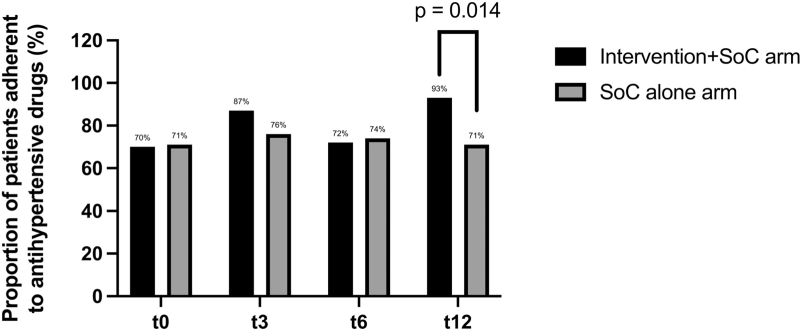
Adherence rate of antihypertensive drugs at four time points. ^a^Only patients with t0, t3, or t6 and t12 are included in this figure.

There was a significant difference in adherence between the SoC and intervention+SoC arm after 12 months of follow-up (93 versus 71%, *P* = 0.014) (Fig. 4). Furthermore, a larger number of patients in the SoC arm became nonadherent during the trial as compared to the intervention+SoC arm (*n* = 7 versus *n* = 1, *P* = 0.086; Table 2). In Table S4 of the supplemental material, an overview is given of the adherence rates divided by adherence, partial adherence, and nonadherence, for the different arms during the four visits of the trial. No relationship was found between the proportion of patients with resistant hypertension (reference = not resistant) and adherence rate of AHDs [odds ratio (OR) = 0.77, 95% CI = 0.27–2.22, *P* = 0.637] with the DDD (OR = 0.56, 95% CI = 0.25–1.25, *P* = 0.259) and randomization arm (OR = 0.89, 95% CI = 0.73–1.09, *P* = 0.155) as covariates.

**TABLE 2:** Change in adherence to antihypertensive drugs between baseline (t0) and 12 months (t12) of follow-up

	Adherent (t0) – Adherent (t12)	Adherent (t0) – Nonadherent^a^ (t12)	Nonadherent (t0) – Adherent (t12)	Nonadherent (t0) – Nonadherent (t12)	*P* [χ^2^ (3)]
SoC arm (*n* = 42) *n* (%)	23 (54.8)	7 (16.7)	7 (16.7)	5 (11.9)	0.086
Intervention+SoC arm (*n* = 40) *n* (%)	27 (67.5)	1 (2.5)	10 (25.0)	2 (5.0)	

*n*, number of patients; SoC, standard of care; t0, baseline; t12, 12 months follow-up. ^a^Nonadherence is defined as the absence of one or more measured antihypertensive drug concentrations in blood used by the patient.

### Posthoc sensitivity analysis

To explain our findings on the difference in adherence but lack of difference in resistant hypertension at t12 between the arms, additional post hoc analyses were performed. Using a higher cut-off value (140 mmHg SBP and/or 90 mmHg DBP) to define resistant hypertension, the proportion of patients with resistant hypertension in the intervention+SoC arm decreased to 50.0% and in the SoC arm to 48.8%. When using this cut-off at baseline the proportion of patients with resistant hypertension differed between the intervention+SoC arm (85% resistant hypertension) and the SoC alone arm (79% resistant hypertension) (*P* = 0.452). We also explored the influence of the method of blood pressure measurement throughout the study, as 24-h ABPMs were only obliged at t0, but preferred at t3, t6, and t12. This resulted in a decrease in 24-h ABPM use in both arms, which were substituted with AOBPs. At t12, 83% of the patients in the intervention+SoC arm had a 24-h ABPM and in the SoC alone arm 75% (*P* = 0.452). The DDD of AHDs decreased in the intervention+SoC arm with an average of −0.1 and increased in the SoC arm with 0.7. This difference was not statistically significant (*P* = 0.059).

### Adherence estimation of physicians at baseline (t0)

Adherence estimations of physicians at t0 (adherent or nonadherent) were compared with the actual nonadherence determined from the measured drug concentrations (*n* = 90). In 31% (*n* = 28) of the patients, the estimations of the physicians were incorrect (Supplemental material Figure S3) of which 36% consisted of nonadherence that was not recognized by the physician.

## DISCUSSION

This multicenter randomized controlled trial included patients with established resistant hypertension based on a 24-h ABPM. We found a significant improvement in both resistant hypertension and blood pressure in the intervention+SoC arm and SoC alone arm. In addition, we observed a significant difference in AHD adherence between the arms after 12 months of follow-up, despite the study being terminated early due to a larger improvement of resistant hypertension in the SoC arm during an interim analysis. Given that most patients had uncontrolled blood pressure for years before participation, it is highly unlikely that the observed improvements would have been achieved without their participation in this study. No differences were determined in the prevalence of resistant hypertension or blood pressure values between the intervention+SoC arm and the SoC arm at t12. Without taking the randomization into account, the overall proportion of patients with resistant hypertension decreased by 34.5% after a follow-up period of at least 12 months. This decrease in resistant hypertension was related to an average decrease in SBP of 12.0 mmHg and DBP of 5.5 mmHg over time, which is a larger decrease compared to most other intervention trials [23]. We showed that participation in our trial improved blood pressure in both study arms which sustained throughout a follow-up period of 12 months, but failed to establish any difference blood pressure and thereby resistant hypertension between the arms. This finding is in concordance with several other randomized trials that tried to improve blood pressure control by improving adherence with different interventions as compared to ours [9,24,25]. Furthermore, these trials also found an improvement in adherence in the intervention arm. Only one trial found a significant difference in blood pressure in favor of the intervention, with a mean difference of 1.5 mmHg SBP between the control (*n* = 423) and intervention (*n* = 460) arm [26]. The findings of this specific trial could indicate that our trial was stopped too early and that an interim analysis after 3 months with 25 patients in each arm resulted in an incorrect conclusion to close the inclusion prematurely.

However, our trial includes interesting findings that can be used to improve adherence research in patients with uncontrolled blood pressure.

First, the Hawthorne effect has to be taken into account when performing adherence research [27]. Because of the participation in the trial, patients could have changed their behavior because they knew they were being monitored.

As we already expected this effect, we tried to take these changes into account in our power calculation including an improvement of blood pressure control in the SoC arm. However, the Hawthorne effect in our trial was larger than anticipated, which can be derived from the fact that one-third of all eligible patients were not included because of well controlled blood pressure in combination with adherence to all measured AHDs. This increased adherence in the excluded patients may be temporary; however, no follow-up to monitor adherence is available to confirm this.

The extent of the Hawthorne effect was also demonstrated in a prospective observational study called RHYME-AD (Resistant HYpertension: Measure Antihypertensive Drugs) [28]. In this study, AHDs in blood were determined in a random blood sample of included patients. The proportion of nonadherent patients in RHYME-AD with resistant hypertension was 10% higher as compared to the randomized patients in the RHYME-RCT trial, suggesting the influence of the Hawthorne effect.

In addition to the Hawthorne effect, our SoC arm also received extra measurements and, for some patients, extra visits as compared to the real SoC. Usually, patients do not have four ABPMs in 1 year combined with DBS sampling. However, it is very difficult to include patients without signing informed consent or subjecting them to study-related measurements. The use of a true SoC group is therefore almost impossible.

Third, our posthoc sensitivity analysis identified some issues that were not expected when designing the study. Part of the patients developed an aversion to 24-h ABPMs. This is unfortunate, as this is the most reliable method to establish true blood pressure in patients. Because of this issue, we had to move to other methods to measure blood pressure, such as AOBP [29]. This could partly explain why no differences in blood pressure were found between the two arms of the study. Also, the apparent larger difference in resistant hypertension in the SoC arm at t12 as compared to the intervention+SoC arm (59 versus 75%) can be explained by the strict cut-off value for fulfilling the definition of resistant hypertension.

### Strengths

One of the most important strengths of the RHYME-RCT trial is that patients were selected based on blood pressure rather than a suspicion of being nonadherent. Because of this, selection bias was minimized and all patients with and without suspicion of nonadherence were included.

Second, to minimize white-coat hypertension, all patients were obliged to do a 24-h ABPM. Approximately, one-third of the patients that went to the eligibility visit had blood pressures below the threshold for participation in the trial, which shows the value of reliable blood pressure measurements. It should be noted that improved adherence also played a role in this large exclusion of patients at t0, as stated previously.

Furthermore, nonadherence was established with an objective and accurate method namely measuring drug concentrations in blood sampled with DBS. False-positive and negative results were minimized by previous validation studies and thereby it was known that all included drugs could be measured up and till 24 h after intake [6,7,16]. The value of this method was also shown with our data on the estimation of adherence versus the measured adherence, where adherence was estimated incorrectly for one-third of the patients.

Participation in the RHYME-RCT trial resulted in a better intake of AHDs and an improvement in blood pressure with an average decrease of 12 mmHg SBP and 5.5 mmHg DBP in the whole study population. These improvements are clinically relevant and cannot be matched by adding more AHDs to a patient's therapy [30]. Because of these improvements, we established that more than half of the patients approached and measured for our trial had pseudo-RH (80 out of 141 patients).

### Limitations

This trial also has some limitations. The inclusion of patients in our trial stopped prematurely due to futility as already explained in the Materials and methods section. Because of this premature study termination, our study is likely to be underpowered. However, as stated before, our interim analysis was possibly too early in the follow-up and not representative of the actual outcome of the trial. Therefore, the use of an interim analysis should be thought through more thoroughly in future trials before executing this analysis.

Second, our method to identify nonadherence has one drawback and that is the absence of all available AHDs in the method. Currently, only 12 out of more than 50 AHDs can be accurately measured with this method. This is mainly due to the validation process. If accurate measurements are needed, the amount of drugs in a method is limited [16]. Also, DBS is not only a convenient sampling method to use but also makes the validation more difficult as less blood is available as compared to plasma from a venipuncture.

### Recommendations

Although we could not prove the effectiveness of our intervention, we assume that attention and communication to establish a behavioral change in a patient do improve medication adherence [31]. Attention and communication do not necessarily result in spending more time and money. Communication tools are freely available in most cases and when done effectively, communication will diminish time spent in the doctor's office [32].

Also, implementation of an accurate and easy-to-use tool like our DBS method to use more regularly at hospital or GPs visits is recommended to support physicians in making clinical decisions about blood pressure control [33]. These regular measurements and feedback as part of SoC make it less delicate to discuss the results and variation in drug concentrations and can also be used to assess adherence over time. Lastly, the combination of measuring drug concentrations with an accurate blood pressure measurement at the same moment in time makes results reliable and white-coat adherence visible.

In conclusion, after 12 months of follow-up, adherence to AHDs in the intervention+SoC arm significantly increased compared with adherence in the SoC arm. However, this did not result in a lower BP or difference in the proportion of resistant hypertension in the intervention+SoC arm compared with the SoC arm.

## ACKNOWLEDGEMENTS

The authors would like to thank dr. A.H. van den Meiracker, prof. E.J. Hoorn, and prof. P.M.L.A. van den Bemt for their contribution to the research protocol. They also would like to thank J.S. Burgerhart, M.W.F. van den Hoogen, M. Lafeber, M. Sonneveld, D. Severs, R. Zietse, A. El Ourouti, L. Commandeur, A. Heida, L.K. Tjong, S. de Koning, H. Nouwen, C. Leunis, N. Maas, B. van Ginneken, H. Jongen, K. Schoenmakers, D. Bartels en B. Ferreira Sousa for their help with the patient inclusion. Lastly, they want to thank our colleagues from the Holter-room for their help with the 24h-ABPMs and all the analysts from the hospital pharmacy laboratory for their help with the DBS measurements.

The data that support the findings of this study are available upon reasonable request from the PI, [JV] following a 12 months embargo from the date of publication to publish additional results from the study (in accordance with our statement at www.zorggegevens.nl).

L.E.J.P. contributed to the study design and optimization of the DBS sampling method, wrote the manuscript, performed research, and analyzed data.

D.A.H., J.B.vdN., S.C.CH., vdL.,M.E.,P.J.G.vdV., I.M.vdM., E.L.E.dB., A.A.K., S.I.-T., N.A.J.vdL. wrote the manuscript and performed research.

S.B. wrote the manuscript, performed research, and contributed to the optimization of the DBS sampling method.

E.B. contributed to the study design, wrote the manuscript, and analyzed data. E.K. Massey and L. van Dijk developed the communication tool, performed research, and wrote the manuscript.

T.vG., M.H.W.K., B.C.P.K. and J.V. designed research, performed research and wrote the manuscript.

This study was supported by a ZonMW grant for Rational Pharmacotherapy (Project number 848016003).

### Conflicts of interest

L.E.J.P. has received lecture fees from Astellas Pharma. D.A.H. has received lecture fees and consulting fees from Astellas Pharma, Astra-Zeneca, Chiesi Pharma, Medincell, Novartis Pharma, Sangamo Therapeutics, and Vifor Pharma. He has received grant support from Astellas Pharma, Bristol-Myers Squibb, and Chiesi Pharma (paid to his institution). D.A.H. does not have employment or stock ownership at any of these companies, and neither does he have patents or patent applications. In the last 3 years, T.vG. has received lecture fees and study grants from Chiesi and Astellas, in addition to consulting fees from Roche Diagnostics, Thermo Fisher, Vitaeris, CSL Behring, Astellas, and Aurinia Pharma. In the last 3 years, L.vD. has received a research grant from Teva for a study not related to this one. The other authors declare no conflicts of interest.

## Supplementary Material

## References

[1] Hyman DJ Pavlik V. Medication adherence and resistant hypertension. *J Hum Hypertens* 2015; 29:213–218. 25209307 10.1038/jhh.2014.73

[2] Judd E Calhoun DA. Apparent and true resistant hypertension: definition, prevalence and outcomes. *J Hum Hypertens* 2014; 28:463–468. 24430707 10.1038/jhh.2013.140 PMC4090282

[3] Zeller A Taegtmeyer A Martina B Battegay E Tschudi P. Physicians’ ability to predict patients’ adherence to antihypertensive medication in primary care. *Hypertens Res* 2008; 31:1765–1771. 18971555 10.1291/hypres.31.1765

[4] Al-Hassany L Kloosterboer SM Dierckx B Koch BC. Assessing methods of measuring medication adherence in chronically ill children-a narrative review. *Patient Prefer Adherence* 2019; 13:1175–1189. 31413546 10.2147/PPA.S200058 PMC6660631

[5] Burnier M. Drug adherence in hypertension. *Pharmacol Res* 2017; 124:1124–1140. 10.1016/j.phrs.2017.08.015 28870498

[6] Peeters LEJ Feyz L Hameli E Zwart T Bahmany S Daemen J. Clinical validation of a dried blood spot assay for 8 antihypertensive drugs and 4 active metabolites. *Ther Drug Monit* 2020; 42:460–467. 31593031 10.1097/FTD.0000000000000703

[7] Peeters Laura EJ Feyz L Boersma E Daemen J van Gelder T Koch Birgit CP. Clinical applicability of monitoring antihypertensive drug levels in blood. *Hypertension* 2020; 76:80–86. 32418497 10.1161/HYPERTENSIONAHA.120.15038

[8] Delavar F Pashaeypoor S Negarandeh R. The effects of self-management education tailored to health literacy on medication adherence and blood pressure control among elderly people with primary hypertension: a randomized controlled trial. *Patient Educ Couns* 2020; 103:336–342. 31451361 10.1016/j.pec.2019.08.028

[9] Hedegaard U Kjeldsen LJ Pottegård A Henriksen JE Lambrechtsen J Hangaard J. Improving medication adherence in patients with hypertension: a randomized trial. *Am J Med* 2015; 128:1351–1361. 26302142 10.1016/j.amjmed.2015.08.011

[10] Morawski K Ghazinouri R Krumme A Lauffenburger JC Lu Z Durfee E. Association of a smartphone application with medication adherence and blood pressure control: the MedISAFE-BP Randomized Clinical Trial. *JAMA Intern Med* 2018; 178:802–809. 29710289 10.1001/jamainternmed.2018.0447 PMC6145760

[11] Gupta P Patel P Štrauch B Lai FY Akbarov A Gulsin GS. Biochemical screening for nonadherence is associated with blood pressure reduction and improvement in adherence. *Hypertension* 2017; 70:1042–1048. 28847892 10.1161/HYPERTENSIONAHA.117.09631 PMC5642335

[12] Brinker S Pandey A Ayers C Price A Raheja P Arbique D. Therapeutic drug monitoring facilitates blood pressure control in resistant hypertension. *J Am Coll Cardiol* 2014; 63:834–835. 24315901 10.1016/j.jacc.2013.10.067 PMC4374549

[13] De Geest S Zullig LL Dunbar-Jacob J Helmy R Hughes DA Wilson IB. ESPACOMP Medication Adherence Reporting Guideline (EMERGE). *Ann Intern Med* 2018; 169:30–35. 29946690 10.7326/M18-0543 PMC7643841

[14] Schulz KF Altman DG Moher D Group C. CONSORT 2010 statement: updated guidelines for reporting parallel group randomised trials. *BMJ* 2010; 340:c332. 20332509 10.1136/bmj.c332 PMC2844940

[15] Peeters LEJ Kappers MHW Boersma E Massey EK van Dijk L van Gelder T. The effect of combining therapeutic drug monitoring of antihypertensive drugs with personalised feedback on adherence and resistant hypertension: the (RHYME-RCT) trial protocol of a multicentre randomised controlled trial. *BMC Cardiovasc Disord* 2023; 23:87. 36788491 10.1186/s12872-023-03114-0 PMC9926861

[16] Peeters LEJ Bahmany S Dekker T Aliawi A van Domburg B Versmissen J. Development and validation of a dried blood spot assay using UHPLC-MS/MS to identify and quantify 12 antihypertensive drugs and 4 active metabolites: clinical needs and analytical limitations. *Ther Drug Monit* 2022; 44:568–577. 35383727 10.1097/FTD.0000000000000984 PMC9275854

[17] Whelton PK Carey RM Aronow WS Casey DE Jr Collins KJ Dennison Himmelfarb C. 2017 ACC/AHA/AAPA/ABC/ACPM/AGS/APhA/ASH/ASPC/NMA/PCNA Guideline for the Prevention, Detection, Evaluation, and Management of High Blood Pressure in Adults: Executive Summary: a report of the American College of Cardiology/American Heart Association Task Force on Clinical Practice Guidelines. *Hypertension* 2018; 71:1269–1324. 29133354 10.1161/HYP.0000000000000066

[18] Williams B Mancia G Spiering W Agabiti Rosei E Azizi M Burnier M. 2018 ESC/ESH Guidelines for the management of arterial hypertension: the Task Force for the management of arterial hypertension of the European Society of Cardiology (ESC) and the European Society of Hypertension (ESH). *Eur Heart J* 2018; 39:3021–3104. 30165516 10.1093/eurheartj/ehy339

[19] Peeters LEJ van der Net JB Schoenmakers-Buis K van der Meer IM Massey EK van Dijk L. Introducing the importance and difficulties of a three-step approach to improve nonadherence to antihypertensive drugs: a case series. *J Hypertens* 2022; 40:189–193. 34857710 10.1097/HJH.0000000000003001 PMC8654304

[20] Groenland EH van Kleef M Bots ML Visseren FLJ van der Elst KCM Spiering W. Plasma trough concentrations of antihypertensive drugs for the assessment of treatment adherence: a meta-analysis. *Hypertension* 2021; 77:85–93. 33249865 10.1161/HYPERTENSIONAHA.120.16061 PMC7720878

[21] Vasbinder EC Goossens LM Rutten-van Mölken MP de Winter BC van Dijk L Vulto AG. e-Monitoring of Asthma Therapy to Improve Compliance in children (e-MATIC): a randomised controlled trial. *Eur Respir J* 2016; 48:758–767. 27230437 10.1183/13993003.01698-2015

[22] Abdulsalim S Unnikrishnan MK Manu MK Alrasheedy AA Godman B Morisky DE. Structured pharmacist-led intervention programme to improve medication adherence in COPD patients: a randomized controlled study. *Res Social Adm Pharm* 2018; 14:909–914. 29104008 10.1016/j.sapharm.2017.10.008

[23] Peacock E Krousel-Wood M. Adherence to antihypertensive therapy. *Med Clin North Am* 2017; 101:229–245. 27884232 10.1016/j.mcna.2016.08.005 PMC5156530

[24] Santschi V Rodondi N Bugnon O Burnier M. Impact of electronic monitoring of drug adherence on blood pressure control in primary care: a cluster 12-month randomised controlled study. *Eur J Intern Med* 2008; 19:427–434. 18848176 10.1016/j.ejim.2007.12.007

[25] van der Laan DM Elders PJM Boons C Nijpels G van Dijk L Hugtenburg JG. Effectiveness of a patient-tailored, pharmacist-led intervention program to enhance adherence to antihypertensive medication: the CATI Study. *Front Pharmacol* 2018; 9:1057. 30319409 10.3389/fphar.2018.01057 PMC6169131

[26] Manel P Carlos B Rafael G Anna A Carmen S Mariano de la F. Multicenter cluster-randomized trial of a multifactorial intervention to improve antihypertensive medication adherence and blood pressure control among patients at high cardiovascular risk (The COM99 Study). *Circulation* 2010; 122:1183–1191. 20823391 10.1161/CIRCULATIONAHA.109.892778 PMC3001186

[27] Davis SA Feldman SR. Using Hawthorne effects to improve adherence in clinical practice: lessons from clinical trials. *JAMA Dermatol* 2013; 149:490–491. 23715042 10.1001/jamadermatol.2013.2843

[28] Peeters LEJ Hesselink DA Lafeber M Severs D van den Hoogen MWF Sonneveld MAH. Monitoring antihypertensive drug concentrations to determine nonadherence in hypertensive patients with or without a kidney transplant. *J Hypertens* 2023; 41:1239–1244. 37195099 10.1097/HJH.0000000000003459 PMC10328507

[29] Peeters LEJ van Oortmerssen JAE Derks LH den Hertog H Fonville S Verboon C. Comparison of automated office blood pressure measurement with 24-h ambulatory blood pressure measurement. *Blood Press* 2022; 31:9–18. 35037533 10.1080/08037051.2021.2013115

[30] Markovitz AA Mack JA Nallamothu BK Ayanian JZ Ryan AM. Incremental effects of antihypertensive drugs: instrumental variable analysis. *BMJ* 2017; 359:j5542. 29273586 10.1136/bmj.j5542 PMC5736968

[31] Zolnierek KB Dimatteo MR. Physician communication and patient adherence to treatment: a meta-analysis. *Med Care* 2009; 47:826–834. 19584762 10.1097/MLR.0b013e31819a5acc PMC2728700

[32] Linn AJ van Weert JC Schouten BC Smit EG van Bodegraven AA van Dijk L. Words that make pills easier to swallow: a communication typology to address practical and perceptual barriers to medication intake behavior. *Patient Prefer Adherence* 2012; 6:871–885. 23271896 10.2147/PPA.S36195 PMC3526884

[33] Berra E Azizi M Capron A Hoieggen A Rabbia F Kjeldsen SE. Evaluation of adherence should become an integral part of assessment of patients with apparently treatment-resistant hypertension. *Hypertension* 2016; 68:297–306. 27296995 10.1161/HYPERTENSIONAHA.116.07464

