# Supplementary figures and images for: Antihypertensive drug concentration measurement combined with personalized feedback in resistant hypertension: a randomized controlled trial
Source: J Hypertens. 2023 Oct 18;42(1):169–78. doi: 10.1097/HJH.0000000000003585 (PMC10713002; doi:10.1097/HJH.0000000000003585)

**Figure S1. Flow chart of sample size calculation.**

**
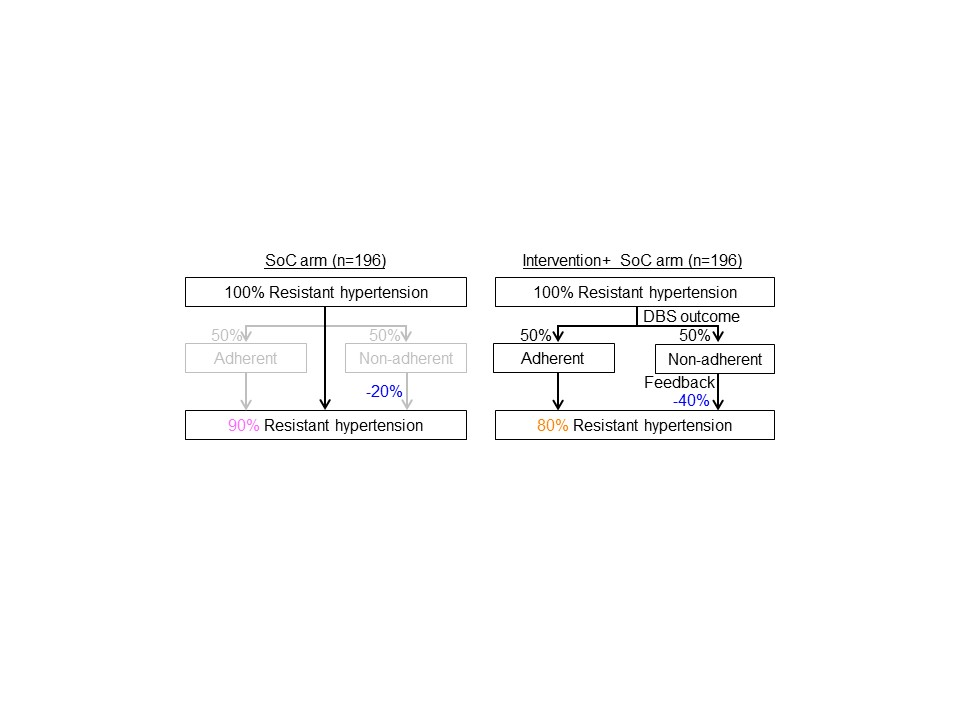
**

Supplement: Supplementary file 1 [file jhype-42-169-s001.doc]
