# Supplementary material for: Antihypertensive drug concentration measurement combined with personalized feedback in resistant hypertension: a randomized controlled trial
Source: J Hypertens. 2023 Oct 18;42(1):169–78. doi: 10.1097/HJH.0000000000003585 (PMC10713002; doi:10.1097/HJH.0000000000003585)
Supplement: Supplementary file 2 [file jhype-42-169-s002.doc]

**Figure S2. Systolic and diastolic blood pressure change between the visits divided by the intervention+SoC and standard of care (SoC) arm.**

**
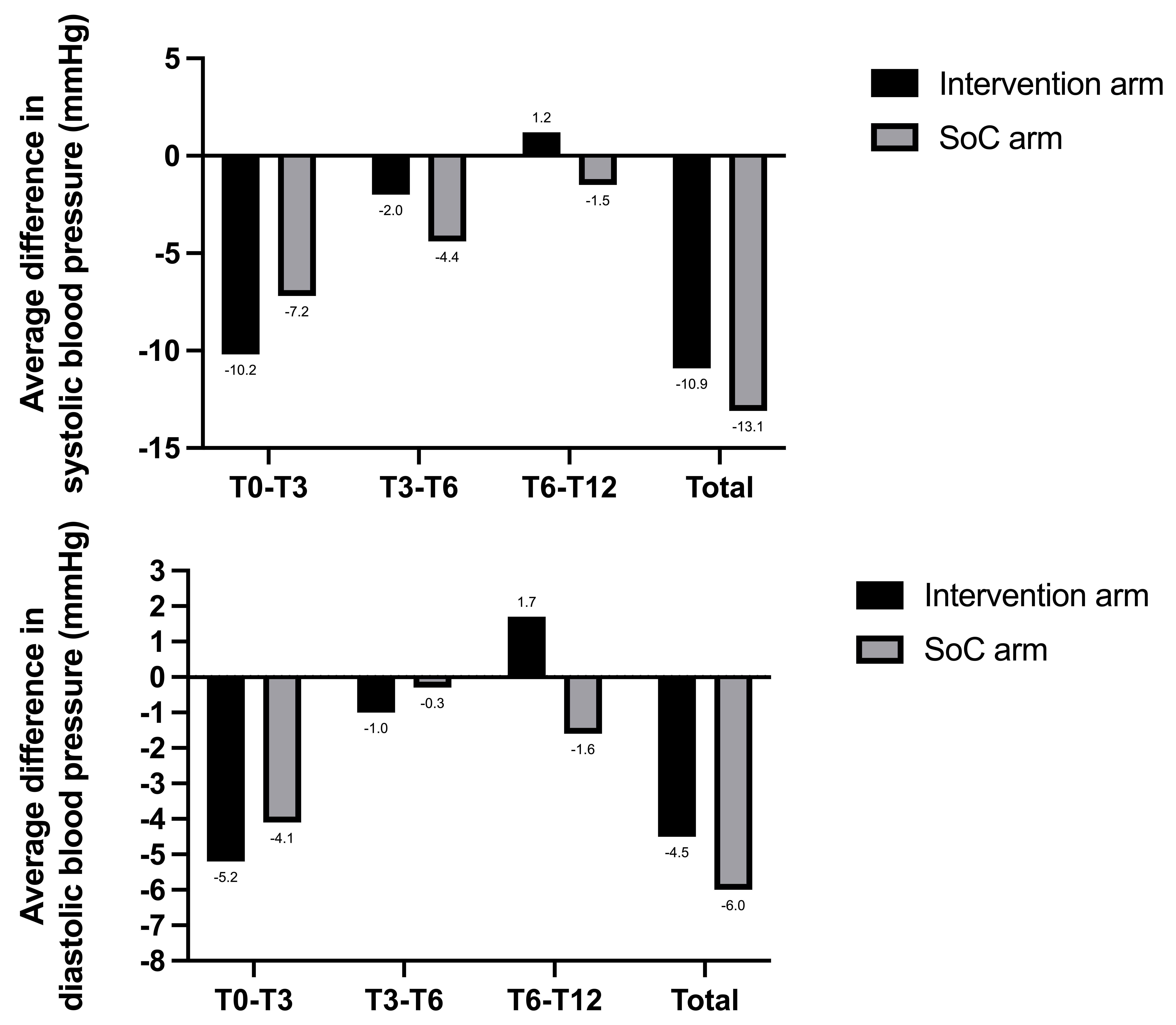
**
