# Supplementary material for: Antihypertensive drug concentration measurement combined with personalized feedback in resistant hypertension: a randomized controlled trial
Source: J Hypertens. 2023 Oct 18;42(1):169–78. doi: 10.1097/HJH.0000000000003585 (PMC10713002; doi:10.1097/HJH.0000000000003585)
Supplement: Supplementary file 3 [file jhype-42-169-s003.doc]

**Figure S3. Adherence estimations by physicians compared to adherence measured by drug concentrations in blood sampled by means of a dried blood spot in patients with confirmed resistant hypertension**

**
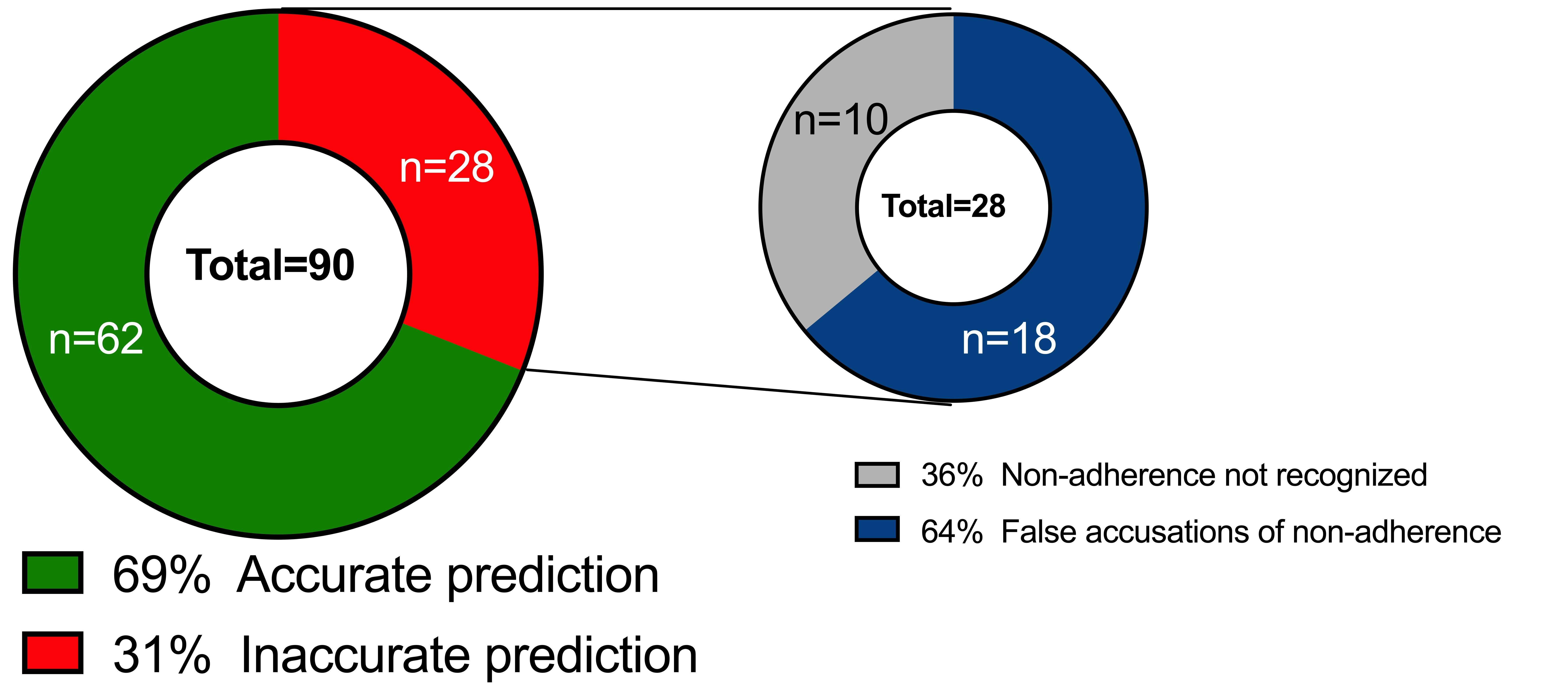
**
