# Supplementary material for: Antihypertensive drug concentration measurement combined with personalized feedback in resistant hypertension: a randomized controlled trial
Source: J Hypertens. 2023 Oct 18;42(1):169–78. doi: 10.1097/HJH.0000000000003585 (PMC10713002; doi:10.1097/HJH.0000000000003585)
Supplement: Supplementary file 4 [file jhype-42-169-s004.docx]

**SUPPLEMENTAL MATERIAL**

|  | | **Adherent,  n (%)** | **Partially adherent, n (%)** | **Non-adherent, n (%)** |
| --- | --- | --- | --- | --- |
| **Visit 1** (t0)** | **Intervention + SoC arm (n=40)** | 28 (70.0) | 10 (25.0) | 2 (5.0) |
|  | **SoC alone arm (n=42)** | 30 (71.4) | 8 (19.0) | 4 (9.5) |
| **Visit 2***  **(t3)** | **Intervention + SoC arm (n=39)** | 34 (87.2) | 5 (12.8) | 0 |
|  | **SoC alone arm (n=41)** | 31 (75.6) | 6 (14.6) | 4 (9.8) |
| **Visit 3***  **(t6)** | **Intervention + SoC arm (n=39)** | 28 (71.8) | 10 (25.6) | 1 (2.6) |
|  | **SoC alone arm (n=38)** | 28 (73.7) | 9 (23.7) | 1 (2.6) |
| **Visit 4**  **(t12)** | **Intervention + SoC arm (n=40)** | 37 (92.5) | 3 (7.5) | 0 |
|  | **SoC alone arm (n=42)** | 30 (71.4) | 10 (23.8) | 2 (4.8) |

Table S4 Adherence to antihypertensive drugs per visit and randomization arm of patients participating in the RHYME-RCT study determined by drug concentrations in blood.

** Patients were allowed to miss either visit 2 or 3.
** The intervention was performed after the adherence measurement of visit 1.*
